# Revisiting the Effect of the Resistance to Gas Accumulation in Constant Volume Systems on the Membrane Time Lag

**DOI:** 10.3390/membranes14080167

**Published:** 2024-07-30

**Authors:** Peter Jr. Leszczynski, Siamak Lashkari, Boguslaw Kruczek

**Affiliations:** 1 Department of Chemical and Biological Engineering, University of Ottawa, Ottawa, ON K1N 6N5, Canada; plesz072@uottawa.ca; 2 Worley Chemetics, Burnaby, BC V5A 4T7, Canada; siamak.lashkari@worley.com

**Keywords:** time-lag method, gas transport properties, constant volume system, poly(phenylene) oxide

## Abstract

The time-lag method is commonly used to determine membrane permeability, diffusivity and solubility in a single gas permeation experiment in a constant volume system. An unwritten assumption on which this method relies is that there is no resistance to gas accumulation in the downstream receiver of the system. However, this is not the case, even with the specially designed receiver used in this study when, in addition to tubing, the receiver utilizes an additional accumulation tank. The resistance to gas accumulation originates from a finite diffusivity (Knudsen diffusion) of gases in tubing, which are magnified by “resistance-free” accumulation tank(s). As a result of the resistance to gas accumulation, the time lag of the membrane is underestimated, which leads to an overestimation of gas diffusivity in the membrane. The experimentally predicted resistances in different configurations of the receiver, expressed by the difference in the time lag at two different receiver locations, were several times greater than the theoretically predicted values. A high molecular PPO membrane was used to demonstrate this effect. The time lags measured at different locations differed by as much as 30%. The diffusivity of nitrogen in a PPO of 4.04 × 10^−12^ m^2^/s determined at the optimum configuration of the receiver is at least 50% lower than the literature-reported values.

## 1. Introduction

The time-lag method, which originated from more than a century-old work of Daynes [1], is the standard approach for evaluating membrane permeability (*P*), diffusivity (*D*) and solubility (*S*) coefficients in a single gas permeation experiment in a constant volume (CV) system. A gas permeation rate (*Q*) measurement in the CV system relies on accurately monitoring pressure rise (*dp*/*dt*) in a known volume (*V*) downstream from the membrane [2]:

(1) $$Q=\frac{V}{RT}\frac{dp}{dt}$$

where *R* is the universal gas constant and *T* is the absolute temperature. A standard experimental protocol in the time-lag method involves first degassing the membrane, i.e., evacuation of the upstream and downstream receivers of the CV system to the highest possible vacuum. Then, the upstream receiver is instantaneously pressurized, and the resulting pressure rise at the permeate side of the membrane is monitored using a high-resolution pressure transducer. The extrapolation of the linear portion of the pressure rise to the time axis yields a time lag (*θ*), which is related to *D* [3]:

(2) $$\theta =\frac{L^{2}}{6D}$$

where *L* is the membrane thickness. At the same time, the slope of the linear portion of the pressure response is directly proportional to the steady-state *Q* from which *P* is determined. In addition, in the solution-diffusion model, the ratio of *P* and *D* represents *S*.

The advantage of having a high initial vacuum in the time-lag technique is three-fold. First, Equation (2) relies on several assumptions, including the initial concentration of the gas in the membrane being nil. From the practical point of view, the resolution of a high-quality absolute pressure transducer is typically 1/10,000 of its maximum reading. Thus, the pressure transducer with a maximum reading of 1 Torr and the corresponding resolution of 0.0001 Torr is available when the membrane is initially at a high vacuum [4]. Finally, the influence of a potential temperature variation during the experiment on the measured *Q* is minimized [5]:

On the other hand, when the permeating gas accumulates at a high vacuum, the mechanism of transport downstream from the membrane is initially Knudsen diffusion. The latter exists when the collisions between gas molecules and the walls of the downstream receiver are much more probable than the collision between the gas molecules, and the corresponding diffusion coefficient (*D_K_*) in cylindrical tubes is given by the following [6]:

(3) $$D_{K}=\frac{2}{3}r\sqrt{\frac{8RT}{\pi M}}$$

where *r* is the radius of the tube and *M* is the molecular weight of the gas. Therefore, the *D_K_* of N_2_ at 300 K in standard 1/2 in. stainless steel tubes, commonly used in downstream receivers of a CV system, is 1.62 m^2^/s, which is very large but not an infinite value.

Although not explicitly stated, the time-lag method assumes that there is no resistance to gas accumulation downstream from the membrane, and the location of the pressure transducer where the pressure rise is monitored is irrelevant. Indeed, the effect of the location of the pressure transducer on the measured *Q* is relatively small [7], but the measured time lag of the membrane could be over or underestimated, which implies that there is a location downstream from the membrane where the time lag could be measured accurately [8]. According to Equation (3), the smaller the tube diameter, the smaller *D_K_* and, consequently, the greater the resistance effects. The downstream receiver of CV systems often contains accumulation tanks [9]. In principle, the internal diameter of the cylindrical tanks for gas accumulation is much greater than that of the tubing, so they can be considered “resistance-free”. However, such a “resistance-free” accumulation tank greatly magnifies the resistance effects in the tube connecting it to the membrane cell [9]. The “resistance-free” tank leads to a negative error in the measured time lag and, in extreme cases, may lead to a negative apparent time lag of the membrane, which is physically impossible. For a given length of the tube, the location of the tank relative to the membrane cell is critical; the further away from the membrane cell, the greater the resistance effects [10]. The analysis of the effect of the “resistance-free” tank in the downstream receiver on the experimental time lag of the membrane was extended to a general case of the receiver consisting of multiple tanks of different volumes located at different positions from the membrane cell [11]. Understanding the resistance to gas accumulation in the CV system allows for designing the configuration of the downstream receiver to minimize the resistance effects and to determine the optimum location of the pressure transducer to monitor the experiment. However, regardless of how poor that configuration is, knowing its details allows for meaningful gas transport properties to be recovered from the apparent pressure rise data [12].

The problem of a lack of standard testing protocol when characterizing gas separation membranes in CV systems has been addressed recently [2]. Yet, despite more than a decade since the last publication related to the resistance to gas accumulation, which likely contributes to the poor repeatability of gas transport properties of the same membrane material tested in different labs, this topic has not generated much interest in the membrane community. This paper aims to re-emphasize the importance of this problem using a configuration of the downstream volume that minimizes the resistance effects. The progress of the time-lag experiments was monitored independently by two absolute pressure transducers, one located close and the other far from the membrane cell. The statistical significance of the contribution arising from the resistance to gas accumulation was investigated. A quantitative and qualitative comparison of the observed resistance effect was compared to the analytical model developed in Ref. [11]

## 2. Theoretical Background

**Figure 1:**
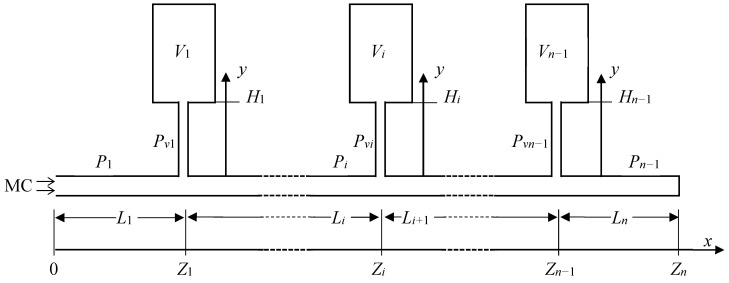
Schematic representation of a multi-tank receiver of a CV system for modelling purposes. Adapted from Ref. [11].

Figure 1 presents a generalized representation of a multiple-tank downstream receiver of a CV system. *n* − 1 cylindrical tanks are connected to the straight mainline (*x*-direction) of length *Z_n_* via *n* − 1 perpendicular connecting tubes located at different distances (*Z_i_*) from the membrane cell. The connecting tubes divide the mainline into *n* segments (parts). The tubes in the mainline may have different lengths (*L_i_*) but have the same internal diameter. The tanks may have different volumes (*V_i_*). Although not shown in Figure 1, each tank can be included or excluded from the downstream volume by respective valves at the entrance to each tank. The distance (*H_i_*) of the tank from the main line, i.e., the length of the connecting tubes, can be different, but all connecting tubes have the same internal diameter. The diameter of the mainline tubes can differ from that of the connecting tubes. At any time, the pressures *p_i_* in the mainline tubes and *p_vi_* in the connecting tubes depend on the distances *x* and *y* from the entrance to the respective tubes.

After initiating the experiment, Fick’s second law of diffusion governs the pressure response in each tube.

(4) $$\frac{\partial p_{i}}{\partial t}=D_{i}\frac{\partial^{2}p_{i}}{\partial x_{i}^{2}}$$

(5) $$\frac{\partial p_{vi}}{\partial t}=D_{vi}\frac{\partial^{2}p_{vi}}{\partial y_{i}^{2}}$$

where the diffusion coefficients in the mainline tubes (*D_i_*) and the connecting tubes (*D_vi_*) are given by Equation (3). Before starting the experiment, the pressure downstream is zero, which represents the initial condition for Equations (4) and (5). At a given time, the pressure at a junction of two or more tubes is the same. Also, the pressure in any tank is the same as the pressure at the junction of the respective tube. In other words, all tanks are assumed to be resistance-free because of their relatively large internal diameter. The expressions for the time lag in the tubes are obtained using the Laplace transforms and the asymptotic solution [11]:

Mainline tubes:

(6) $$\frac{\theta_{i}}{\theta_{0}}=\frac{\gamma }{\theta_{0}}+\frac{\Psi }{\theta_{0}}\;-3{\left(\frac{Z_{n}-x}{Z_{n}}\right)}^{2}-6\sum_{j=i}^{n-1}\left(\frac{A_{Vj}H_{j}+V_{j}}{A_{i}Z_{n}}\right)\left(\frac{Z_{j}-x}{Z_{n}}\right)$$

Connecting tubes:

(7) $$\frac{\theta_{vi}}{\theta_{0}}=\frac{\theta_{i}\left(x=Z_{i}\right)}{\theta_{0}}+3\frac{D_{i}}{D_{vi}}\frac{H_{i}\left(H_{i}+2\frac{V_{i}}{A_{vi}}\right)}{Z_{n}^{2}}-3\frac{D_{i}}{D_{vi}}\left({\left(\frac{H_{i}-y_{i}}{Z_{n}}\right)}^{2}+2\frac{V_{i}}{A_{vi}Z_{n}}\frac{\left(H_{i}-y_{i}\right)}{Z_{n}}\right)$$

where *A_i_* and *A_vi_* are the internal cross-section areas of the mainline and connecting tubes, respectively, and

(8) $$\theta_{0}=\frac{Z_{n}^{2}}{6D_{i}}$$

(9) $$\frac{\theta_{i}\left(x=Z_{i}\right)}{\theta_{0}}=\frac{\gamma }{\theta_{0}}+\frac{\Psi }{\theta_{0}}\;-3{\left(\frac{Z_{n}-Z_{i}}{Z_{n}}\right)}^{2}-6\sum_{j=i+1}^{n-1}\left(\frac{A_{vj}H_{j}+V_{j}}{A_{i}Z_{n}}\right)\left(\frac{Z_{j}-Z_{i}}{Z_{n}}\right)$$

(10) $$\begin{array}{ll} \frac{\gamma }{\theta_{0}} & =\frac{A_{i}Z_{n}}{V_{tl}}-6\sum_{j=1}^{n-1}\frac{D_{i}A_{vj}H_{j}}{D_{vj}V_{t}}\left(\frac{1}{3}{\left(\frac{H_{j}}{Z_{n}}\right)}^{2}+\frac{V_{j}H_{j}}{A_{vj}Z_{n}^{2}}+{\left(\frac{V_{j}}{A_{vj}Z_{n}}\right)}^{2}\right)\\ & +3\sum_{j=1}^{n-1}\left(\frac{A_{vj}H_{j}}{V_{t}}+\frac{V_{j}}{V_{t}}\right)\left(\frac{Z_{j}^{2}+{\left(Z_{n}-Z_{j}\right)}^{2}}{Z_{n}^{2}}\right) \end{array}$$

(11) $$\frac{\Psi }{\theta_{0}}=6\frac{V_{t}}{A_{i}Z_{n}}\sum_{j=1}^{n-1}\left(\frac{A_{vj}H_{j}+V_{j}}{V_{t}}\right)\sum_{k=j+1}^{n-1}\left(\frac{A_{vk}H_{k}+V_{k}}{V_{t}}\right)\left(\frac{Z_{k}-Z_{j}}{Z_{n}}\right)$$

where *V_t_* is the total volume of the downstream receiver.

## 3. Materials and Methods

### 3.1. Materials

High-molecular-weight PPO powder with a molar mass of 350,000 g/mol was supplied by SABIC (Sittard, The Netherlands). To fabricate the membrane, PPO was dissolved in trichloromethane (MilliporeSigma Canada Ltd., Oakville, ON, Canada, 480150) using a magnetic stirrer over 48 h to create a 7.5% *w*/*w* polymer solution. The PPO solution was cast on a silicon wafer using a spin coater under a vacuum at 150 rpm for 5 min. A second layer was cast on top of the first using the same PPO solution at 150 rpm for 5 min under vacuum. After spin coating, the silicon wafer-backed PPO films were transferred to a vacuum desiccator for 48 h, after which the PPO films were liberated from the wafers by immersion in deionized water. The PPO films were then dried between multiple sheets of paper and pressed flat by an 8-inch concrete block (Home Depot, Kanata, ON, Canada, 30169112). The PPO films were left between the paper for two months before being cut into a round disc. The average thickness of the PPO membrane was 47.5 µm.

### 3.2. CV System and the Experimental Protocol

**Figure 2:**
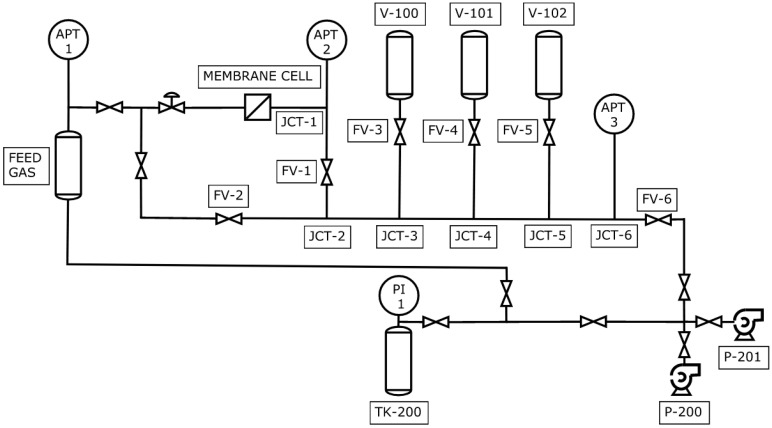
A schematic of the constant volume (CV) system that was used in this study. APT = absolute pressure transducer, FV = manually operated stainless steel bellow sealed valves, TK = compressed gas (nitrogen) cylinder, P = rotary or turbomolecular pump, V = accumulation tank, PI = pressure regulator, JCT = junction.

Figure 2 presents a schematic diagram of the CV system used in this study. After installing the membrane inside the cell, the system was extensively evacuated to degas the membrane using a rotary vacuum pump P-200 (CANVACTECH, Ottawa, ON, Canada, Edwards RV3, A65201906, ultimate pressure: 1.5 × 10^−3^ Torr) and a turbomolecular pump P-201 (Edwards Vacuum LLC Sanborn, New York, NY, USA, Edwards TS85D1002, ultimate pressure < 3.75 × 10^−10^ Torr). A freshly installed membrane required five days until degassing could no longer be detected. Between the tests, one day of evacuation was sufficient for complete degassing. Before each experiment, a leak test was performed when the system was at a high vacuum, 10^−4^ Pa. The leak rate, determined from the pressure rise recorded by two identical 10-Torr absolute pressure transducers APT2 and APT3 (CCR Process Products Kanata, Ontario, Canada, MKS627F11TBC1B, 10^−5^ full-scale resolution, accuracy: 0.12% of reading, range: 0 to 10 Torr), never exceeded 8.1 × 10^−8^ Pa/s. The system was pressurized to 1600 Torr using a nitrogen gas cylinder TK-200 (99.998% purity, Linde Mississauga, ON, Canada), and the upstream pressure was monitored with the absolute pressure transducer APT1 (CCR Process Products Kanata, ON, Canada, MKS627F13TBC1B, 10^−5^ full-scale resolution, accuracy: 0.12% of reading, range: 0 to 2000 Torr). The FV valves in Figure 1 were manually operated stainless steel bellow sealed valves (Swagelok SC-4BK-VCR). They were procured from Weston Valve & Fitting LTD, Mississauga, ON (Canada). With FV-2 closed (i.e., the upstream and downstream volumes separated), the time-lag experiments were initiated by opening the valve separating the membrane cell from the upstream volume.

The progress of the experiment was monitored independently by the APT2 and APT3 located at 0.095 m and 0.519 m from the membrane cell. Closing the FV-1 valve, located 0.165 m from the membrane cell, would allow for minimizing the volume of the downstream receiver to *V_t_* = 2.84 × 10^−6^ m^3^, which would be essential when testing barrier materials. However, to investigate the effects of the resistance to gas accumulation experimentally, time-lag experiments were performed with FV-1 open. The valves in the downstream receiver were manually operated stainless steel bellow sealed valves (Swagelok SC-4BK-VCR) procured from Weston Valve & Fitting LTD, Mississauga, ON, Canada). The total volume of the downstream receiver was modulated by adding/removing the accumulation tanks, V-100 (0.3 × 10^−3^ m^3^), V-101 (0.5 × 10^−3^ m^3^) and V-102 (0.5 × 10^−3^ m^3^) to the downstream receiver. The respective distances of these tanks from the membrane cell were 0.302 m, 0.367 m and 0.432 m. Because the APT2, APT3, V-100, V-101 and V-102 had 3/8-inch fittings, the tubing between the membrane cell and FV-1 and that between FV-3, FV-4, FV-5 and the respective accumulation tanks were standard SS 3/8-inch OD tubes. All other tubing, including the mainline between FV-1 and FV-6 and the connecting tubes up to FV-3, FV-4 and FV-5, were standard SS 1/2-inch OD tubes.

The time-lag experiments were performed in four different downstream configurations (R_i_). The details of the configuration are listed in Table 1. The basic configuration (R_1_) includes only tubing in the downstream receiver. The configurations R_2_–R_4_ include one or two accumulation tanks. It is important to emphasize that the downstream receiver in Figure 2 could allow eight different configurations. However, those listed in Table 1 are sufficient to illustrate the effects of the resistance to gas accumulation.

### 3.3. Determination of the Time Lag

**Figure 3:**
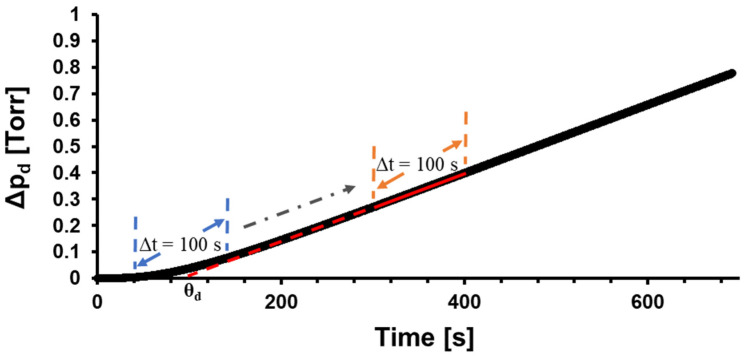
Illustration of the moving window analysis on downstream time-lag experiment.

**Figure 4:**
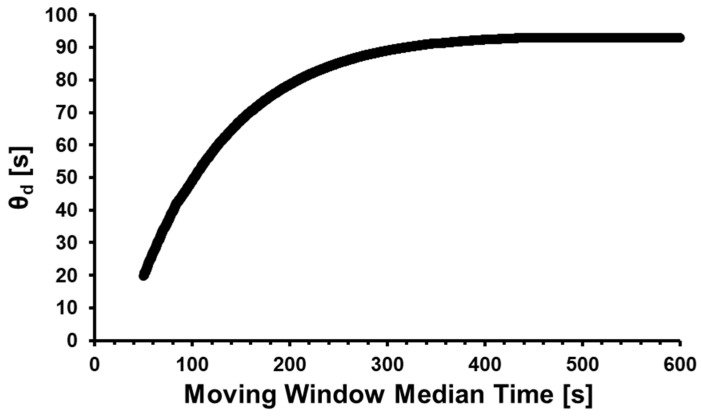
Instantaneous time lag as a function of median moving window time, depicting nitrogen permeating through a PPO membrane with receiver configuration R_1_ at a pressure of 1600 Torr and 303.15 K.

The time lag (*θ*) is commonly determined from the intercept of the extrapolated linear portion of the pressure response with the time axis. Because of gas accumulation downstream from the membrane, the actual steady-state conditions are never reached, and what appears as a linear pressure response signifies pseudo-steady-state conditions. Consequently, the value of *θ* depends on the selection of the range of the pressure response data. In this study, we used the concept of a moving window to avoid the ambiguity associated with commonly determined *θ* values [13]. This concept is demonstrated in Figure 3. The moving window in Figure 3 has a fixed size of 100 s and contains approximately 1700 data points. A linear regression of pressure response within the window’s bounds (solid red line) is extrapolated to the time axis (dashed red line), and the resulting intercept represents an instantaneous time lag (*θ_d_*) for the time from the middle of the window. The window is then shifted one datum at a time, and the new *θ_d_* is obtained. Plotting such determined instantaneous time lag values versus time yields Figure 4.

An inspection of Figure 4 shows that a plateau in calculated instantaneous time-lag values is reached at around a moving window median time of 400–600 s, indicative of a quasi-steady state permeation rate. At this plateau, at a median window time of 400 s, *θ_d_*, *P*, *D* and *S* were determined.

### 3.4. Statistical Analysis

A two-factor experiment with repeated measures was performed so that any interactions or main effects of the receiver configuration and the measurement location could be identified [14]. Factor A was the receiver configuration. Four levels of factor A, i.e., the configurations R_1_–R_4_ (Table 2), were tested. The second factor, factor B, was the measurement location and was tested at two levels using independent absolute pressure transducers, APT2 and APT3 were located 0.095 and 0.519 m downstream from the membrane, respectively. Both levels of factor B were measured simultaneously for each factor A level. The dependent variable studied was the time lag measured by both APTs, and three samples were observed per combination of factors A and B.

The two-factorial test conducted with *i* = 4 levels of factor A and *j* = 2 levels of factor B with *k* = 3 repeated measures of the independent observations for a given trial may be described by the linear statistical model Equation (12) [14].

(12) $$Y_{ijk}=\mu +\tau_{i}+\beta_{j}+{\left(\tau \beta \right)}_{ij}+\varepsilon_{ijk}\left\{\begin{array}{l} i=1,2,3,4\\ j=1,2\\ k=1,2,3 \end{array}\right.$$

where *µ* is the overall mean effect, *τ_i_* is the treatment effect of the *i*th level of factor A, *β_j_* is the treatment effect of the *j*th level of factor *β*, (*τβ*)*_ij_* is the interaction effect between factors A and B at the *i*th and *j*th levels, and *ε_ijk_* is the random error for the *k*th repeat measure at the *i*th and *j*th factor levels.

A two-factor analysis of variance (ANOVA) with repeated measures was performed to determine if factor A or B had a statistically significant effect on the mean time lag. Because each combination of factors A and B had three independent observations, the degrees of freedom allowed for the interaction of factors A and B to be tested. The proposed three null hypotheses were as follows:

1. The effects of each level of factor A on the observed time lag are equal to zero.
2. The effects of each level of factor B on the observed time lag are equal to zero.
3. The effect of the interaction between factors A and B on the observed time lag equals zero.

Or, symbolically, each respective alternative hypothesis is as follows:

(13) $$\begin{array}{l} H_{0}:\tau_{1}=\tau_{2}=\tau_{3}=\tau_{4}=0\\ H_{1}:\;\mathrm{at}\;\mathrm{least}\;\mathrm{one}\;\tau_{i}\neq 0 \end{array}$$

(14) $$\begin{array}{l} H_{0}:\beta_{1}=\beta_{2}=\beta_{3}=\beta_{4}=0\\ H_{1}:\;\mathrm{at}\;\mathrm{least}\;\mathrm{one}\;\beta_{i}\neq 0 \end{array}$$

(15) $$\begin{array}{l} H_{0}:{\left(\tau \beta \right)}_{11}={\left(\tau \beta \right)}_{12}\ldots ={\left(\tau \beta \right)}_{ij}\ldots ={\left(\tau \beta \right)}_{42}=0\\ H_{1}:\;\mathrm{at}\;\mathrm{least}\;\mathrm{one}\;{\left(\tau \beta \right)}_{ij}\neq 0 \end{array}$$

The significance level, *α*, was selected to be 0.01.

Model adequacy was assessed through the analysis of residuals. The normality of the residuals was examined using a quantile normal plot, and homoscedasticity was analyzed by plotting residuals by factor level [14].

## 4. Results

After collecting the raw data, residuals were analyzed to verify the assumptions and applicability of the general linear model and ANOVA. A plot of the cumulative probability vs. residuals showed linear behaviour except for outliers from data collected during the third repeated measure of time lag for the factor A level R_3_. Disregarding the one significant outlier (R_3_ Trial 3), a normal distribution of residuals was observed, satisfying the assumption made. The residuals were further analyzed by factor levels, and equal variance was observed for all factors, except for the factor A level R_3,_ where the outlier was observed in the third repeated measurement, justifying the assumptions of equal variance made. If more than three repeated measures were performed for each combination of factors and levels, the deviations from residual normality and equal variance resulting from the outlier would decrease.

Despite one outlier in the factor A level R_3,_ a two-factor ANOVA with repeated measures was performed, including all levels of factor A (R_3_ was not removed from the data set post hoc). With α = 0.01, the interaction of factors A and B was found to be statistically significant, in addition to the main effects of both factors. Thus, all three null hypotheses were rejected. If the same analysis were performed, removing the factor A level R_3_, the ANOVA analysis would result in the same conclusion but with even greater statistical significance. The ANOVA summary table for the study of the effect of receiver configuration and measurement location on the measured time lag is presented in Table 2.

**Figure 5:**
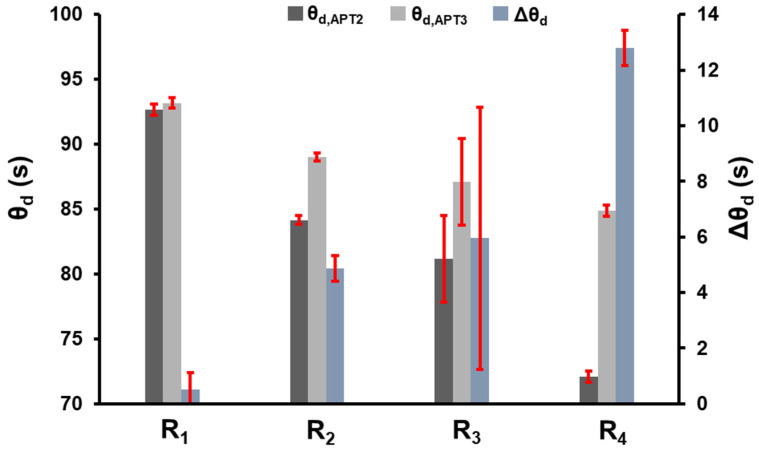
The average time-lag values for receiver configurations R_1_–R_4_. Error bars represent the 95% confidence interval.

The rejection of the null hypothesis for factors A and B indicates that the value of the observed variable, *θ*, depends on the receiver configuration and measurement location. The practical implication is that the transport properties of membranes determined using the time-lag method in a CV system have an inherent error. The significance of the interaction between receiver configuration and measurement location implies that the spatial variation of the resistance to gas accumulation within the receiver is affected by the configuration (total volume and tank locations) utilized. Figure 5 presents the mean observed time lag for each factor and level to investigate the interaction between the two factors further. The same data shown in Figure 5 are provided in Table 3 for reference.

An inspection of Figure 5 shows that the difference between the mean time-lag values observed by APT2 and APT3 is lowest for receiver configuration R_1_, and the difference increases as additional volumes are added between the two pressure transducers. The mean time-lag difference increases from 0.52 ± 0.58 s for configuration R_1_ to 12.79 ± 0.63 s for configuration R_4_. Furthermore, the measured time lags decrease at both locations as the volume between APT2 and APT3 increases. Although the time lags at both locations decrease with the addition of accumulation tanks, a more significant decrease is observed at the pressure transducer located closest to the membrane cell before additional volumes (APT2) were added, and a lesser effect is observed by the pressure transducer further from the membrane cell after all additional volumes (APT3) were added.

The receiver’s time-lag contribution was simulated for the apparatus outlined in Figure 2 in configurations R_1_–R_4_ at the location of APT2 and APT3 using Equations (6)–(11) [11]. Table 4 summarizes and compares the theoretical predictions to the experimentally observed differences.

## 5. Discussion

The observed time lags (Figure 5 and Table 3) combine the time lag of the membrane and the receiver. Consequently, the receiver’s contribution could not be directly measured. However, by taking the difference between the observed time lag measured by APT3 and APT2, the time-lag contribution of the membrane is removed, and the resulting time-lag differential (Δ*θ*) can be attributed to the receiver alone. Therefore, we will consider Δ*θ* as the measure of the resistance to gas accumulation in the downstream receiver. Table 4 compares the experimental and theoretical values of Δ*θ* in the R_1_–R_4_ receiver configuration.

It is evident that the most straightforward permeate receiver configuration, R_1_, comprises mostly 12.7 mm (1/2 inch) OD tubing with no additional volumes, was predicted to have a time lag contribution of −0.09 s and 0.04 s at the locations of APT2 and APT3, respectively. The corresponding Δ*θ* of 0.13 s represents the lowest resistance of all considered configurations of the downstream receiver, which is the consequence of following the design heuristics proposed in Ref. [11]. More specifically, the mainline utilizes an oversized diameter tubing (1/2 inch OD); the length of the mainline (0.602 m) is minimized for the number of additional accumulation tanks; and most importantly, R_1_ is not using additional accumulation tanks. It is important to emphasize that although the experimental Δ*θ* = 0.52 s was significantly greater than the theoretical value, the effects of resistance to gas accumulation in the R_1_ configuration can be considered negligible, in particular in comparison to the R_2_–R_4_ configurations, i.e., those including at least one accumulation tank.

Because the PPO membrane tested in this study was relatively thick (47.5 µm), the characterization of this membrane would not require an accumulation tank in the downstream receiver. However, because the gas permeation rate is inversely proportional to the membrane thickness if the PPO membrane was 10–15 μm or the membrane was made from a more permeable polymer, the volume of the downstream receiver would need to be increased by adding an accumulation tank to allow a linear pressure response to be reached before the upper limit of the absolute pressure transducer was reached. In other words, the use of the R_2_–R_4_ configuration of the receiver would be required rather than optional. In addition, using a larger volume of the receiver minimizes the magnitude of the pressure rise downstream, which is advantageous from the perspective of the nil concentration at the permeate interface of the membrane on which Equation (2) relies [15]. Because the problems associated with the resistance to gas accumulation did not get across the membrane community, a researcher conducting the time-lag experiment could choose a larger downstream volume (if such flexibility existed) when characterizing their membrane.

Compared to other configurations, R_1_ is the only one for which the theoretical *θ* at APT3 is positive. The negative theoretical *θ* in all other configurations is qualitatively consistent with the experimental time lags. As shown in Table 3, the experimental time lags at APT3 and APT2 in R_1_ are significantly greater than those in other configurations. In other words, the presence of the accumulation tanks in the downstream receiver leads to depression (underestimation) of the time lag of the membrane. It can be shown that if the downstream receiver was a straight cylindrical tube, the combined time lag would simplify to the following [8]:

(16) $$\theta =\theta_{m}+\frac{Z_{n}^{2}}{6D_{i}}-\frac{{\left(Z_{n}-x\right)}^{2}}{2D_{i}}$$

where *θ_m_* is the actual time lag of the membrane. Equation (16) implies that for *x*/*Z_n_* < 0.423, the experimental time lag underestimates *θ_m_*, while the opposite is true for *x*/*Z_n_* > 0.423. Therefore, *x*/*Z_n_* = 0.423 represents the location of the pressure transducer, at which *θ_m_* would be measured accurately regardless of how high the resistance to gas accumulation might be. Because the R_1_ configuration is closest to the ideal straight tube of the downstream receiver, the theoretical *θ* at the APT3 location is a low positive value. The presence of accumulation tank(s) not only underestimates the time lag of the membrane but also moves the location where *θ_m_* is measured accurately further away from the membrane cell [9,11]. As indicated previously, the APT3 was located 0.519 m from the membrane cell, corresponding to *x*/*Z_n_* = 0.86. Yet, the maximum theoretical *θ* at APT3 is −0.60 s for the R_2_ configuration of the receiver. Although −0.60 s might appear negligible, the resistance effects must be much more significant because the experimental time at APT3 in R_2_ is more than 4 s lower than in R_1_.

The model given by Equations (6)–(11) is qualitatively consistent with the experimental results. However, it significantly underestimates the effects of the resistance to gas accumulation in the downstream receiver. In other words, the problem associated with the resistance to gas accumulation is much worse than what is predicted by Equations (6)–(11). The critical assumption when deriving these equations is that T-connections, tube bends, adapters, and open valves do not impose any resistance to gas accumulation [11]. The measure of these additional resistances is provided by the difference between the experimental and theoretical Δ*θ* of the R_1_ configuration, i.e., 0.52 s compared to 0.13 s. Without any additional accumulation tanks, these resistances are negligible as they are < 1 s. However, adding accumulation tanks magnifies these otherwise negligible resistances. Equations (6)–(11) predict this effect well. For example, the theoretical Δ*θ* in R_2_ is nearly 5 times and, in R_4_, nearly 13 times that in R_1_.

The absolute theoretical value of the time lag in the R_1_ at APT3 is smaller than at APT2. Consequently, the experimentally measured time lag by APT3 in the R_1_ configuration of 93.18 s is the closest to the actual time lag of the tested PPO membrane. Considering the experimental Δ*θ* and the theoretical *θ*s at APT2 and APT1 in the R_1_ configuration, it can be concluded that the actual time lag of the membrane is approximately 93 s, i.e., *θ* at APT3 in R_1_ is slightly overestimated, but not by more than 0.2 s. We will consider *θ_m_* = 93.13 s as a reference value in the following discussion.

As previously stated, the attractiveness of the time-lag method arises from the ability to determine *D*, *P* and *S* from a single gas permeation experiment. More specifically, *D* is evaluated from the time lag using Equation (2), *P* from the slope of the linear pressure response, and *S* from the ratio of *P* and *D*. Table 5 summarizes the transport properties of nitrogen in PPO based on experimental results collected by APT2 and APT3 in four different configurations of the downstream receiver. In addition to the absolute values of *D*, *P* and *S*, the relative error in these transport properties using the properties based on APT3 in R_1_ as the reference values is also provided.

Comparing the relative errors between the possible configurations and measurement locations, it is evident that the time-lag contribution of the receiver has the most significant effect on the *D*, which was overestimated anywhere from 0.56% to 29.27%, depending on the pressure transducer and configuration used. The overestimation of *D* is a direct consequence of the underestimation of time lag. In turn, because *P* was affected by the configuration and the location of the pressure transducer a little, the overestimation of *D* led to an underestimation of *S* calculated from the ratio of *P* and *D*.

A high molecular PPO is a common membrane material for gas separation membranes, and the nitrogen transport properties in the PPO from the literature converted to units consistent with those in Table 5 are summarized in Table 6. The conditions at which the reported *P*, *D* and *S* were determined are also provided. The most reliable *P*, *D* and *S* from this study (based on APT3 in the R_1_ configuration) are also listed for comparison. Although most references listed in Table 6 utilized a CV system to characterize their membranes, they do not provide any details about the configuration of their downstream receiver.

It can be noticed that the nitrogen transport properties in Table 6 vary from reference to reference. The different conditions at which these properties were determined partly contribute to this variation. For example, the temperature in Table 6 varies from 22 to 35 °C. It is generally known that *P* and *D* increase while *S* decreases with temperature [28]. Although the variation in pressures in Table 6 is even greater than that of temperature, the effect of pressure on *P*, *D* and *S* is not expected to be as significant as that of temperature. The gas transport properties in polymeric membranes depend on the molecular weight of the polymer; more specifically, *P* increases with the molecular weight [20,22]. While the effect of molecular weight on *P* is relatively modest, the degree of crystallinity profoundly affects *P*, *D* and *S*, which all increase with the degree of crystallinity [27]. PPO is a semicrystalline polymer containing both amorphous and crystalline phases, and the degree of crystallinity, which is generally low, depends on the source of the polymer and the conditions at which the membranes were prepared [29].

Despite the variation in the testing conditions, molecular weight and the degree of crystallinity of PPOs in Table 6, it can be noticed that the diffusivity of nitrogen in this study is the lowest of all values reported in the literature. The latter values are even greater than 5.23 × 10^−12^ m^2^/s, i.e., the largest diffusivity from Table 5, which is overestimated by the effects of the resistance to gas accumulation. Because the references from Table 6, which reported the diffusivity of nitrogen in PPO, did not provide any details about the configuration of their downstream receiver, it cannot be concluded that the resistance to gas accumulation was responsible for the higher literature values. Still, at the same time, this possibility cannot be ruled out. Equations (6)–(11) indicate that a relatively small resistance in the mainline of the receiver is greatly magnified by the presence of the accumulation tank(s). This study used an oversized 1/2-inch OD mainline tube to minimize the resistance effects. If a standard 1/4-inch OD tube was used, the resistance effects in R_2_–R_4_ configurations would significantly increase compared to those reported in Table 4.

## 6. Conclusions

We have systematically studied the effects of the resistance to gas accumulation in different configurations of the downstream receiver of a constant volume on the time lag of a PPO membrane. The receiver was designed to minimize the resistance effects and simultaneously allow the characterization of a wide range of membrane materials by changing the receiver volume in different system configurations depending on the anticipated permeation rate. A difference between the time lags close to the membrane cell and close to the end of the receiver’s mainline represented a measure of the resistance to gas accumulation. The experimentally determined resistances were compared to theoretically predicted values. This comparison revealed that the experimental values are several-fold higher than the theoretical ones. These differences were due to neglecting the resistance to gas accumulation arising from some necessary features of the downstream receiver, such as T-connections, tube bends and open valves unaccounted for in the theoretical model.

Although the resistance effects are independent of the tested membranes, we used the experimental data collected simultaneously at two locations within the receiver to evaluate nitrogen diffusivity, permeability and solubility in the PPO membrane. Because the resistance effects in the configuration without an additional accumulation tank were less than one second, the properties from this configuration were considered the closest to the actual values. Including an accumulation tank in the downstream receiver led to underestimating the time lag and consequently overestimating the diffusivity up to 30%. Interestingly, the literature diffusivities of nitrogen in PPO are significantly greater than that of 30%. Because the configurations of downstream receivers are not revealed in the literature, it cannot be concluded if the observed difference resulted from the resistance to gas accumulation. Although underestimation of the membrane time lag is more prevalent close to the membrane, it could not be avoided even at the dimensionless distance of 0.86 from the membrane cell.

## Figures and Tables

**Table 1:** Valve settings in the downstream receiver in different system configurations.

Configuration	Open Valves	Closed Valves	Volume [m^3^]
R_1_	FV-1	FV-2,3,4,5,6	8.1 × 10^−5^
R_2_	FV-1,3	FV-2,4,5,6	3.8 × 10^−4^
R_3_	FV-1,4	FV-2,3,5,6	5.8 × 10^−4^
R_4_	FV-1,3,4	FV-2,5,6	8.8 × 10^−4^

**Table 2:** Two-factor ANOVA with repeated measures summary table for determining the statistical significance of the time-lag contribution of the downstream receiver.

Source of Variation	SS	DF	MS	F	*p*-Value	F Crit.
Factor A	642.98	3	214.33	108.31	7.69 × 10^−11^	5.29
Factor B	220.59	1	220.59	111.47	1.28 × 10^−8^	8.53
Interaction	115.81	3	38.60	19.51	1.36 × 10^−5^	5.29
Within	31.66	16	1.98			
Total	1011.05	23				

**Table 3:** Mean time-lag values for receiver configurations R_1_–R_4_.

Downstream Receiver Configuration	*θ* (APT2) [s]	*θ* (APT3) [s]
R_1_	92.66	93.18
R_2_	84.15	89.01
R_3_	81.15	87.10
R_4_	72.08	84.87

**Table 4:** Predicted receiver time-lag contributions for configurations R_1_–R_4_ and the experimentally observed time-lag differential between APT3 and APT2.

Downstream Receiver Configuration	Simulated			Experimental
	*θ* (APT2) [s]	*θ* (APT3) [s]	Δ*θ* [s]	Δ*θ* [s]
R_1_	−0.09	0.04	0.13	0.52 ± 0.58
R_2_	−1.21	−0.60	0.61	4.86 ± 0.47
R_3_	−2.32	−1.16	1.16	5.94 ± 4.72
R_4_	−2.66	−1.04	1.63	12.79 ± 0.63

**Table 5:** The nitrogen transport properties *P*, *D* and *S* of the PPO membranes determined using receiver configurations R_1_–R_4_ and the relative errors to R_1_ APT3.

Downstream Receiver Configuration	Transport Properties			Relative Error		
	*P* mol·m/s·m^2^·Pa	*D* m^2^/s	*S* mol/m^3^·Pa	*P* %	*D* %	*S* %
	**APT2**					
R_1_	1.08 × 10^−15^	4.07 × 10^−12^	2.66 × 10^−4^	1.42	0.56	0.85
R_2_	1.09 × 10^−15^	4.48 × 10^−12^	2.43 × 10^−4^	1.92	10.73	−7.95
R_3_	1.09 × 10^−15^	4.65 × 10^−12^	2.35 × 10^−4^	2.32	14.96	−10.90
R_4_	1.10 × 10^−15^	5.23 × 10^−12^	2.11 × 10^−4^	3.50	29.27	−19.93
	**APT3**					
R_1_	1.07 × 10^−15^	4.04 × 10^−12^	2.64 × 10^−4^	0.00	0.00	0.00
R_2_	1.07 × 10^−15^	4.23 × 10^−12^	2.53 × 10^−4^	0.58	4.68	−3.91
R_3_	1.08 × 10^−15^	4.33 × 10^−12^	2.49 × 10^−4^	1.20	7.01	−5.41
R_4_	1.09 × 10^−15^	4.44 × 10^−12^	2.45 × 10^−4^	1.91	9.78	−7.17

**Table 6:** The nitrogen transport properties *P*, *D* and *S* in the PPO from the literature.

*P* mol·m/s·m^2^·Pa	*D* m^2^/s	*S* mol/m^3^·Pa	Comments	Ref.
1.17 × 10^−15^	6.68 × 10^−12^	4.41 × 10^−4^	@1.5 atm and 35 °C	[16]
0.87 × 10^−15^	8.41 × 10^−12^	1.04 × 10^−4^	@30 °C	[17]
1.57 × 10^−15^	-	-	@100 kPa and 25 °C	[18]
1.34–2.01 × 10^−15^	-	-	@100 kPa and 25 °C; *P* increases with MW of PPO	[19]
1.00–1.30 × 10^−15^	-	-	@6.58 atm and 22–24 °C; *P* increases with MW of PPO	[20]
0.44 × 10^−15^	-	-	@1 atm and 25 °C	[21]
1.56 × 10^−15^	-	-	@30 °C	[22]
1.27 × 10^−15^	8.41 × 10^−12^	1.59 × 10^−4^	@300–900 kPa	[23]
1.04 × 10^−15^	-	-	@4–8 atm and 22–23 °C	[24]
1.22 × 10^−15^	7.80 × 10^−12^	1.56 × 10^−4^	@300–900 kPa and 25 °C	[25]
1.00 × 10^−15^	-	-	@1300 kPa and 35 °C	[26]
1.04–5.36 × 10^−15^	6.40–11.0 × 10^−12^	1.61–5.02 × 10^−4^	@0.7–0.9 atm and 35 °C; *P*, *D*, *S* increase with the degree of crystallinity of PPO	[27]
1.07 × 10^−15^	4.04 × 10^−12^	2.64 × 10^−4^	@2.11 atm and 30 °C	This work

## Data Availability

The raw data supporting the conclusions of this article will be made available by the authors on request.
